# Role of handgrip strength in predicting new-onset diabetes: findings from the survey of health, ageing and retirement in Europe

**DOI:** 10.1186/s12877-021-02382-9

**Published:** 2021-07-29

**Authors:** Guochen Li, Yanan Qiao, Yanqiang Lu, Siyuan Liu, Yi Ding, Xing Chen, Chaofu Ke

**Affiliations:** 1 Department of Epidemiology and Biostatistics, School of Public Health, Medical College of Soochow University, 215123 Suzhou, China; 2 Department of Preventive Medicine, College of Clinical Medicine, Suzhou Vocational Health College, 215009 Suzhou, China; 3 Department of Children Health Care Affiliated, Suzhou Hospital of Nanjing Medical University, No.26, Dao Qian Road, 215000 Suzhou, China

**Keywords:** Handgrip strength, Predictive ability, Diabetes, SHARE

## Abstract

**Background:**

Diabetes is a major concern for the global health burden. This study aimed to investigate the relationship between handgrip strength (HGS) and the risk of new-onset diabetes and to compare the predictive abilities between relative HGS and dominant HGS.

**Methods:**

This longitudinal study used data from the Survey of Health, Ageing and Retirement in Europe (SHARE), including 66,100 European participants aged 50 years or older free of diabetes at baseline. The Cox proportional hazard model was used to analyze the relationship between HGS and diabetes, and the Harrell’s C index, net reclassification index (NRI), and integrated discrimination improvement (IDI) were calculated to evaluate the predictive abilities of different HGS expressions.

**Results:**

There were 5,661 diabetes events occurred during follow-up. Compared with individuals with lowest quartiles, the hazard ratios (95 % confidence intervals) of the 2nd-4th quartiles were 0.88 (0.81–0.94), 0.82 (0.76–0.89) and 0.85 (0.78–0.93) for dominant HGS, and 0.95 (0.88–1.02), 0.82 (0.76–0.89) and 0.60 (0.54–0.67) for relative HGS. After adding dominant HGS to an office-based risk score (including age, gender, body mass index, smoking, and hypertension), the incremental values of the Harrell’s C index, NRI, IDI of relative HGS were all slightly higher than those of dominant HGS in both training and validation sets.

**Conclusions:**

Our findings supported that HGS was an independent predictor of new-onset diabetes in the middle-aged and older European population. Moreover, relative HGS exhibited a slightly higher predictive ability than dominant HGS.

## Background

Diabetes has long been a major concern for global health systems [1]. As obesity rates rise, the population ages and urbanization accelerates, the prevalence of diabetes continues to increase [2]. According to the latest version of the International Diabetes Federation (IDF), the global prevalence of diabetes is estimated to be 9.3 % (463 million people) in 2019 and will rise to 10.9 % (700 million people) by 2045 [2]. In addition, about 4.2 million adults aged 20–79 years died of diabetes globally in 2019, with the global direct healthcare expenditure for diabetes estimated at $760 billion in the same year [3, 4]. Furthermore, the growth rate of diabetes in Western European countries is higher than the global average [5]. These unfavorable statistics highlight that diabetes remains a serious public health issue, which calls for effective prevention and control measures.

Handgrip strength (HGS), as a simple anthropological measurement and an indicator of upper body muscle strength, is considered to be related to insulin action and the risk of the onset and death of diabetes [6–8]. Strong muscle strength can effectively increase the content of glucose transporter and various myokines, which regulate glucose metabolism and insulin resistance [9, 10]. Data from several longitudinal cohort studies involving adults in the US, UK, and China have shown that low HGS is positively associated with the risk of diabetes [6, 11]. However, the results were not totally consistent. The Prospective Urban-Rural Epidemiology (PURE) study reported that there was no association between HGS and the incidence of diabetes after surveying 17 countries with different income levels [12]. Moreover, the CoLaus (Cohorte Lausannoise) study came to the same conclusion with PURE after applying the multivariate adjustment model [13]. In addition, varied HGS measurement methods, including dominant HGS and relative HGS, were applied to explore their associations with diabetes. There are studies pointed out that relative HGS might have an advantage in predicting the risk of cardiovascular biomarkers, metabolic profile, and other cardiometabolic disorders [14–16]. Moreover, a Basque study emphasized the need for more in-depth research to help determine whether relative or absolute indicators have more practical applications [17]. Therefore, the real relationship between HGS and diabetes and the predictive potential of different HGS expressions on diabetes risk remain to be clarified.

In the present study, we explored the longitudinal association between HGS and diabetes based on a nationally representative cohort study of middle-aged and older adults across Europe. In addition, we also assessed and compared the predictive values of different HGS expressions on diabetes risk.

## Methods

### Study design and population

The present study used data from the Survey of Health, Ageing and Retirement in Europe (SHARE). The respondents of SHARE are individuals over the age of 50 and their spouses or partners in 29 European countries [18]. The survey began in 2004, and the main goal was to provide information about demographics, income, assets, health, cognition, family structure and relationships, health care use and costs, and expectations. Participants were then followed up approximately every 2 years. Details of the study design of SHARE can be found in previous literatures [18, 19]. SHARE has obtained approval from the Ethics Council of the Max Planck Society and Ethics Committee of the University of Mannheim, and all participants signed an informed consent form.

**Fig. 1:**
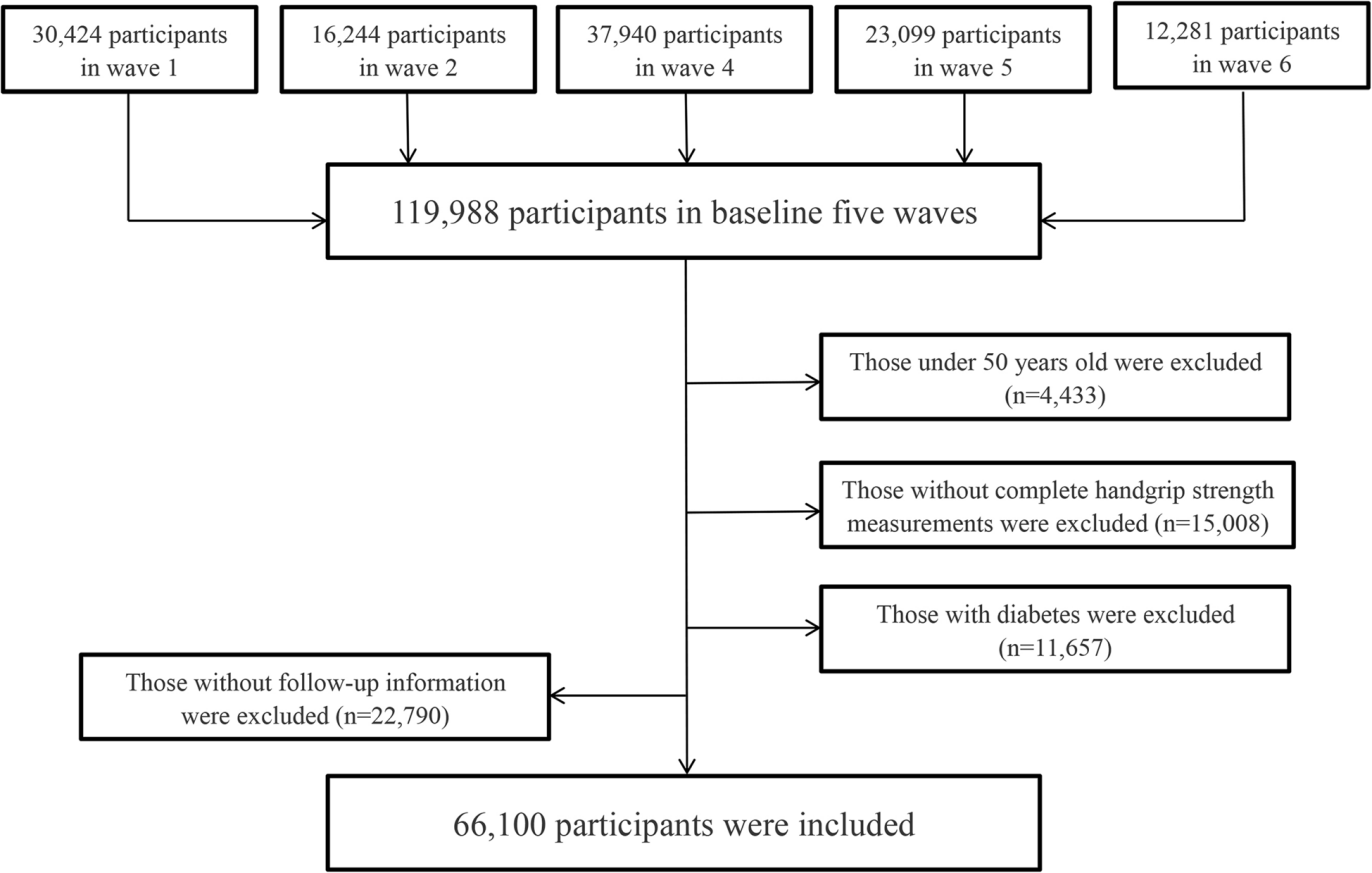
The flow chart of the selection of the study population

We excluded the respondents who did not meet all of the following criteria at baseline: (1) aged at least 50; (2) successful measurement of HGS; (3) not suffering from diabetes; (4) at least one complete follow-up record. Finally, 66,100 participants in SHARE were eligible for subsequent analysis. The detailed screening procedure of study subjects was shown in Fig. 1.

### Measurement of HGS

HGS was measured with the aid of the same type of dynamometer (Smedley, S Dynamometer, TTM, Tokyo, 100 kg) [20]. Before the measurement, participants were asked if they were in a safe state. If the respondents had surgery, any swelling, inflammation, severe pain, or injury on one or both hands in the past six months or refused to accept the measurement, the measurement was given up. The test was performed with elbows at 90°angles on both sides in a standing (preferably) or sitting position. Keeping the wrist in a neutral position and adjusting the inner lever of the dynamometer to fit the hand. Both hands were measured alternately twice. In the present study, the body mass index (BMI, kg/m^2^) was defined as the weight (kg) divided by the square of height (m). Dominant HGS was regarded as the maximal value between two measurements of the dominant hand, and absolute HGS was defined as the sum of the maximum HGS values measured by both hands. Relative HGS was computed as absolute HGS divided by BMI [21, 22]. The HGS data we used were all measured at baseline when participants entered the study.

### Assessment of new-onset diabetes

In this study, the diagnosis of diabetes was ascertained from two aspects. On the one hand, participants were asked whether a doctor has told them they had or currently has diabetes or high blood sugar at the baseline survey and each follow-up. On the other hand, the respondents would be asked to reply whether they are taking any medication for diabetes right now. If one of the affirmative answers was given, they were regarded as diabetes cases. The new-onset diabetes cases were defined as those developing diabetes during follow-up but without diabetes history at baseline. And we also determined the onset time of diabetes from two aspects: (1) If the age first diagnosed with diabetes was reported, the age was defined as the onset time of diabetes; (2) If the age first diagnosed with diabetes was unavailable, we used the midpoint of the interval between the previous wave and the latest wave with diagnosing information as the onset time. The included participants in our study needed to have complete diabetes data from at least two time points (in addition to the baseline record, an extra follow-up time point was required).

### Covariates

The covariates in this study included sociodemographic characteristics, lifestyle factors, and self-reported history of chronic diseases. Sociodemographic characteristics covered age (years, continuous), gender (man/woman), residence, country, and self-reported education attainment. The residence was classified as urban vs. rural. Education attainment was divided into three levels: less than upper secondary education, upper secondary and vocational education, tertiary education. Lifestyle factors included self-reported drinking and smoking status. Smoking status was categorized as never smoking and ever smoking. Drinking was classified as drinking and non-drinking during the last 7 days. BMI (kg/m^2^) was incorporated in a continuous manner. Self-reported history of chronic diseases included high blood pressure, cancer, lung disease, heart problems, stroke, arthritis, high cholesterol, Parkinson’s disease, hip fracture, and ulcer. History of chronic diseases was recorded at baseline and classified as YES vs. NO. The aim of including both common and rare chronic diseases was to eliminate the effects of known and potential confounding factors wherever possible.

### Statistical analysis

In the baseline survey, the Wilcoxon rank sum test was applied to compare continuous data, and the chi-square test was used to compare categorical data. In the main analysis, the relationship between HGS (in quartiles or continuous forms) and diabetes was investigated following three steps.

First, we used the Cox proportional hazards regression model to estimate the hazard ratio (HR) and 95 % confidence interval (95 % CI) for the association between HGS and diabetes in SHARE. We ran a total of four models: model 0 (unadjusted) only included HGS indicators; Model 1 was adjusted for age and gender; Model 2 was adjusted for office-based risk factors (such as age, gender, BMI, hypertension, and smoking) which is often used to predict cardiovascular and other health events [23]; Model 3 was adjusted for all potential confounders, including age, gender and country, residence, education, BMI, smoking, and drinking status, history of chronic diseases.

Second, to validate the predictive ability of HGS, we randomly divided the whole dataset into training and validation sets of equal size, with stratification by gender to ensure the same ratio of men and women in both sets. The Harrell’s C index, net reclassification index (NRI), and integrated discrimination improvement (IDI) were calculated in both sets to evaluate the incremental predictive values of dominant HGS and relative HGS for new-onset diabetes beyond office-based risk factors [23–25]. We set the truncation time at the median follow-up time in both sets [24].

Finally, we presented the main results using the combined quartiles (synthesizing standards for men and women) because HGS levels differ significantly between the two genders. In addition, we investigated whether there were interactions between HGS and age or gender, and then performed stratified analyses by gender and age (< 60 years old or ≥ 60 years old).

The statistical analysis was performed using SAS 9.4 (SAS Institute Inc, Cary, NC, USA) and R 4.0.1. Two-sided *P* < 0.05 was deemed as statistically significant.

## Results

Among 66,100 participants included in this study, a total of 5,661 individuals developed diabetes during follow-up. The incidence of diabetes was 15.1 per 1000 person-years. Participants who suffered from diabetes tended to be older and male, possessed higher BMI, and had lower education levels (all *P* < 0.01). The prevalence of hypertension, lung problems, arthritis, high cholesterol, and ulcer in the diabetes group was significantly higher than that in the non-diabetic group (*P* < 0.01) (Table 1).

**Table 1:** Characteristics of the participants at baseline

Characteristics	Non-diabetes	Diabetes	*P* value
N	60,439 (91.44)	5,661 (8.56)	-
Age (years)	61 (15)	64 (14)	<0.01
Male (%)	26,996 (44.67)	2,789(49.27)	<0.01
Education I (%)	23,592 (39.03)	2,889 (51.03)	<0.01
Education II (%)	22,891 (37.87)	1,909 (33.72)	
Education III (%)	13,956 (23.09)	863 (15.24)	
BMI (kg/m2)	25.88 (5.15)	28.26 (5.78)	<0.01
Current smoke (%)	11,851 (19.64)	1,096 (19.39)	0.65
Current drink (%)	30,368 (50.33)	2,306 (40.79)	<0.01
Rural (%)	24,182 (30.36)	1,517 (28.05)	<0.01
Dominant HGS (kg)	-	-	-
Men	44 (14)	43 (15)	<0.01
Women	27 (9)	25 (10)	<0.01
Relative HGS (m^2^)	-	-	-
Men	3.21 (1.17)	2.89 (1.00)	<0.01
Women	2.00 (0.81)	1.71 (0.74)	<0.01
Heart problems (%)	6,047 (10.01)	909 (16.06)	<0.01
Stroke (%)	1,748 (2.89)	229 (4.05)	<.001
Hypertension (%)	26,988 (32.43)	2,732 (48.26)	<0.01
Lunge problems (%)	2,995 (4.96)	361 (6.38)	<0.01
Cancer (%)	3,390 (5.51)	301 (5.32)	0.36
Arthritis (%)	12,271 (20.30)	1,308 (23.11)	<0.01
High cholesterol (%)	12,131 (20.07)	1,612 (28.48)	<0.01
Parkinson’s disease (%)	236 (0.39)	34 (0.60)	0.02
Hip fracture (%)	1,112 (1.84)	126 (2.23)	0.04
Ulcer (%)	3,537 (5.85)	393 (6.94)	<0.01

Note: Education I — Less than upper secondary; Education II — Upper secondary and vocational; Education III — Tertiary education. Values were presented as n (%), median (interquartile range). Dominant HGS: maximum HGS of the dominant hand; Relative HGS: the sum of the maximum HGS of both hands divided by BMI

Based on the unadjusted model, compared with the lowest quartile, the HR (95 % CI) values of the 2nd-4th quartiles of dominant HGS were 0.72 (0.67–0.77), 0.65 (0.60–0.70) and 0.60 (0.55–0.64), respectively; and as expressed by relative HGS, the HR (95 % CI) values were 0.69 (0.65–0.74), 0.48 (0.45–0.51) and 0.26 (0.24–0.29), respectively. If adjusted by age and gender, similar associations were exhibited. The HR (95 % CI) values of the 2nd-4th quartiles of dominant HGS for developing diabetes were 0.79 (0.74–0.85), 0.75 (0.70–0.81) and 0.74 (0.69–0.81) compared to the lowest quartile. And for relative HGS, they were 0.71 (0.67–0.76), 0.50 (0.46–0.54) and 0.28 (0.25–0.31), respectively. In the model adjusted for office-based risk factors (age, gender, BMI, smoking and hypertension), compared with the bottom quartile, the HR (95 % CI) values of the 2nd-4th quartiles of dominant HGS were 0.81 (0.75–0.87), 0.75 (0.69–0.81) and 0.71 (0.66–0.77), and they were 0.88 (0.82–0.94), 0.72 (0.66–0.78) and 0.49 (0.44–0.54) for relative HGS (Table 2).

**Table 2:** Associations of two HGS expressions with follow-up diabetes

		Dominant HGS					Relative HGS				
	Gender	Q1	Q2	Q3	Q4		Q1	Q2	Q3	Q4	
	Men	<37	37 to 44	44 to 51	≥51	Continuous (Per SD)	<2.6	2.6 to 3.2	3.2 to 3.7	≥3.7	Continuous (Per SD)
	Women	<22	22 to 27	27 to 31	≥31		<1.6	1.6 to 2.0	2.0 to 2.4	≥2.4	
Model 0		1	0.72 (0.67-0.77)	0.65 (0.60-0.70)	0.60 (0.55-0.64)	0.93 (0.91-0.96)	1	0.69 (0.65-0.74)	0.48 (0.45-0.51)	0.26 (0.24-0.29)	0.73 (0.71-0.75)
Model 1		1	0.79 (0.74-0.85)	0.75 (0.70-0.81)	0.74 (0.69-0.81)	0.85 (0.82-0.89)	1	0.71 (0.67-0.76)	0.50 (0.46-0.54)	0.28 (0.25-0.31)	0.56 (0.54-0.58)
Model 2		1	0.81 (0.75-0.87)	0.75 (0.69-0.81)	0.71 (0.66-0.77)	0.83 (0.80-0.86)	1	0.88 (0.82-0.94)	0.72 (0.66-0.78)	0.49 (0.44-0.54)	0.73 (0.70-0.76)
Model 3		1	0.88 (0.81-0.94)	0.82 (0.76-0.89)	0.85 (0.78-0.93)	0.92 (0.88-0.96)	1	0.95 (0.89-1.02)	0.82 (0.76-0.89)	0.60 (0.54-0.67)	0.81 (0.77-0.85)

Note: Q1=the first quartile (lowest); Q2=the second quartile; Q3= the third quartile; Q4= the fourth quartile; Dominant HGS: maximum HGS of the dominant hand; Relative HGS: the sum of the maximum HGS of both hands divided by BMI. Model 0: unadjusted; Model 1 was adjusted for age and gender. Model 2 was adjusted for the office-based risk factors (age, gender, BMI, smoking, and hypertension); Model 3 was adjusted for age, gender, residence, country, BMI, education, drinking and smoking, high blood pressure, cancer, lung disease, heart problems, stroke, arthritis, high cholesterol, Parkinson’s disease, hip fracture, and ulcer

After applying the multi-variable adjustment model (model 3), we observed a positive association between low HGS and diabetes. Compared with the bottom quartile, the HR (95 % CI) values of the 2nd-4th quartiles of dominant HGS were 0.88 (0.81–0.94), 0.82 (0.76–0.89), and 0.85 (0.78–0.93), respectively. As measured by relative HGS, HR (95 % CI) values of the 2nd-4th quartiles for diabetes were 0.95 (0.88–1.02), 0.82 (0.76–0.89) and 0.60 (0.54–0.67), respectively. In addition, we treated HGS as continuous variables and calculated HR (95 % CI) values per standard deviation through the above four models, and all associations were statistically significant (all *P* < 0.01, Table 2).

The capacities of the two HGS expressions for predicting new-onset diabetes were further evaluated. The addition of dominant HGS and relative HGS to the office-based risk score significantly improved the discriminatory power. In the training set, the Harrell’s C statistics were significantly higher for dominant HGS (0.6845, 95 % CI: 0.6744–0.6947) and for relative HGS (0.6872, 95 % CI: 0.6770–0.6973) than that for the office-based risk score (0.6800, 95 % CI: 0.6699–0.6903). The increases in NRI after the office-based risk score were 5.63 % (95 % CI: 2.80–8.21 %) for dominant HGS and 9.75 % (95 % CI: 7.47–12.60 %) for relative HGS; and the augmenters in IDI upon the office-based risk score were 0.15 % (0.06–0.28 %) for dominant HGS and 0.35 % (0.22–0.51 %) for relative HGS. The results of the validation set were all consistent with those of the training set (Table 3). The incremental values of Harrell’s C index, NRI, IDI of relative HGS were all slightly higher than those of dominant HGS in both training and validation sets.

**Table 3:** Reclassification and discrimination statistics for new-onset diabetes by HGS

	Training set							Validation set				
	Harrell’s C index		NRI		IDI		Harrell’s C index		NRI		IDI	
	Estimate (95% CI)	*P*	Estimate (95% CI)	*P*	Estimate (95% CI)	*P*	Estimate (95% CI)	*P*	Estimate (95% CI)	*P*	Estimate (95% CI)	*P*
Office-based risk factors	0.6802 (0.6699-0.6903)	-	-	-	-	-	0.6837 (0.6737-0.6938)	-	-	-	-	-
Plus dominant HGS	0.6845 (0.6744-0.6947)	<0.01	0.0563 (0.0280-0.0821)	<0.01	0.0015 (0.0006-0.0028)	<0.01	0.6877 (0.6778-0.6976)	<0.01	0.0442 (0.0180-0.0678)	<0.01	0.0013 (0.0004-0.0024)	<0.01
Plus relative HGS	0.6872 (0.6770-0.6973)	<0.01	0.0975 (0.0747-0.1260)	<0.01	0.0035 (0.0022-0.0051)	<0.01	0.6899 (0.6800-0.6998)	<0.01	0.0883 (0.0635-0.1143)	<0.01	0.0038 (0.0025-0.0059)	<0.01

Note: office-based risk factors included age, gender, BMI, hypertension, and smoking

**Fig. 2:**
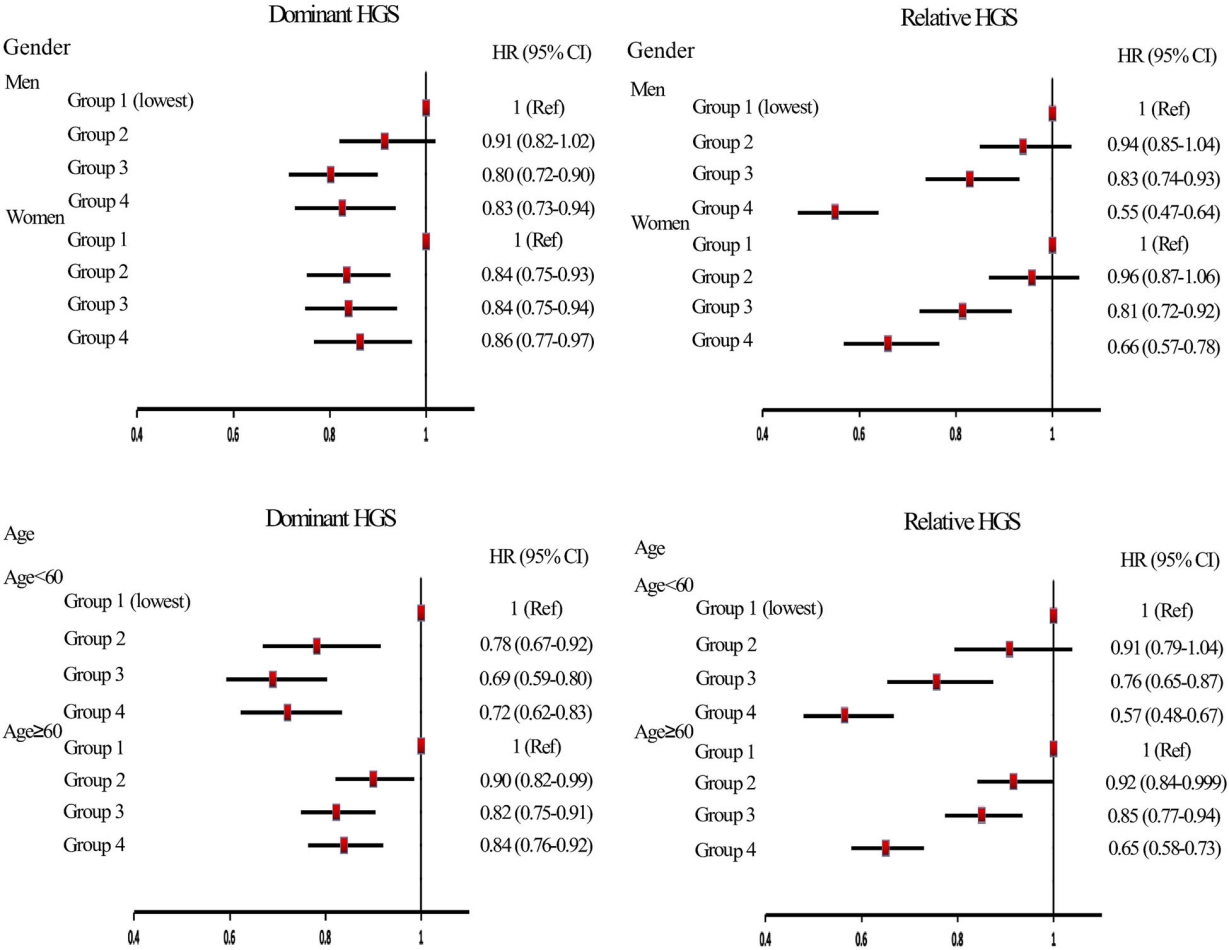
Stratified analyses by gender and age for the association between baseline HGS and follow-up diabetes (Note: Group 1 = the first quartile (lowest); Group 2 = the second quartile; Group 3 = the third quartile; Group 4 = the fourth quartile)

We fitted the HGS*gender and HGS*age interaction terms to our multi-variable model to investigate whether the associations differed with gender or age. And we found no significant interactions for the effects of two HGS expressions on diabetes by gender (both *P* > 0.05). Among men, compared to the lowest quartiles, the HR (95 % CI) values of the 2nd-4th quartiles of dominant HGS for diabetes were 0.91 (0.82–1.02), 0.80 (0.72–0.90), and 0.83 (0.73–0.94), respectively. When we used relative HGS, HR (95 % CI) values decreased, which were 0.94 (0.85–1.04), 0.83 (0.74–0.93) and 0.55 (0.47–0.64) for the 2nd-4th quartiles, respectively. Among women, individuals in the highest quartile of dominant HGS possessed 0.86 (0.77–0.97) times the risk of diabetes than that in the lowest quartile, and the risk was 0.66 (0.57–0.78) times when expressed by relative HGS. The *P* values were 0.3092 for the interaction between dominant HGS and age, and 0.0018 for the interaction between relative HGS and age. Among individuals aged younger than 60, compared to the lowest quartiles, the HR (95 % CI) values of the 2nd-4th quartiles of dominant HGS for diabetes were 0.78 (0.67–0.92), 0.69 (0.59–0.80), and 0.72 (0.62–0.83), respectively. When measured by relative HGS, the risks were 0.91 (0.79–1.04), 0.76 (0.65–0.87) and 0.57 (0.48–0.67) for the 2nd-4th quartiles, respectively. Among people over 60, participants in the highest quartile of dominant HGS possessed 0.84 (0.76–0.92) times the risk of diabetes than that in the lowest quartile, and the risk was 0.65 (0.58–0.73) times when expressed by relative HGS (Fig. 2).

## Discussion

Through a prospective cohort study involving 29 European countries, we found the dose-response relationship between baseline HGS and future diabetes: the higher the HGS level, the lower the risk of diabetes. In addition, the ability of relative HGS to predict new-onset diabetes was slightly higher than that of dominant HGS.

The negative association between HGS and diabetes has been widely reported in previous studies [26–30]. A research based on the Korea National Health and Nutrition Examination Survey (KNHANES) suggested that diabetes was inversely related to HGS in younger women and men [30]. A similar phenomenon was observed in the older population in the UK as well [29]. Likewise, we also observed that there was a clear negative relationship between increasing HGS and the risk of diabetes among middle-aged and older people across Europe. However, there were some exceptions. For example, the PURE study reported that HGS was not significantly associated with the incidence of diabetes in low- and middle-income countries [12]. This study did not include populations from major European countries, and the follow-up time was only four years. Pedro Marques-Vidal et al. also demonstrated that there was no association between HGS and incident type 2 diabetes mellitus in healthy adults. However, only 62.6 % of participants were included from the initial cohort, resulting in a relatively small sample size (*N* = 2,318) [13].

In addition, a study from the UK Biobank involving 418,656 participants pointed out that whether HGS was expressed in absolute or relative values, lower HGS was associated with a higher risk of diabetes after adjusting for factors such as age and education [31]. Although this study involved a European population, it was a cross-sectional study and did not further compare the classification potential of different HGS measurements. Ho et al. pointed out that the relationship between HGS and health outcomes would not be altered by changing how it is expressed, but the endpoint events in this study did not include new-onset diabetes [23]. Moreover, several studies have also shown that higher relative HGS rather than dominant or absolute HGS was associated with lower cardiometabolic risk [14, 15, 32]. Remarkably, our research introduced the Harrell’s C index, NRI, and IDI to compare the abilities of two HGS expressions in predicting diabetes. The results of the three statistics were consistent in our study, indicating that relative HGS exhibited a slightly higher predictive ability than dominant HGS. One of the possible reasons for this phenomenon may be that relative HGS can adjust the degree of the quality confounding and accompanying health risks of increased body size compared with dominant HGS, and the tight link between body type and diabetes has been reported in many studies [33–35].

The potential mechanisms for the connection between HGS and diabetes remain to be clarified. One of the possible mechanisms is that individuals with stronger muscle strength have increased insulin action and lower blood sugar, which may help reduce the risk of diabetes [11]. Muscle strength training has been shown to increase the protein content of glucose transporter (GLUT-4) and improve insulin resistance [9, 36]. Moreover, a variety of myokines (e.g., IL-6) were secreted by contractile skeletal muscle to regulate glucose metabolism [10, 37]. In addition, some studies have pointed out that HGS is closely related to metabolic profiles, markers, and prediabetes [15, 16, 37]. For example, muscle strength, reflected by HGS, is related to metabolic markers such as HbA1c, which is originally an auxiliary diagnostic indicator of diabetes [38].

This study used a large-scale population cohort to investigate the association between HGS and the risk of diabetes. To the best of our knowledge, this study is the first to compare the predictive values of different HGS expressions on future diabetes. However, some limitations in this study are worth noting. First, the definition of chronic diseases uses self-reported information. However, a report involving 26,162 patients verified the coherence between self-reported and actual medical information [39]. Second, since the hematology information of the participants was not available, the diagnosis of diabetes was limited to the self-reported history of diabetes and medication information, which might underestimate the new-onset diabetes cases. Nevertheless, the self-reported doctor’s diagnosis of diabetes has been proven to be reasonably effective [40]. Third, some individuals were excluded due to incomplete information on baseline HGS or diabetes, which may introduce potential bias and limit the generalisability of our findings to some extent. Finally, when the accurate onset time of some diabetes patients was unavailable, we approximated the onset time using the midpoint time. Although this method has been adopted by many studies [41, 42], the accuracy of the onset time could not be fully guaranteed and the measurement error might be introduced.

## Conclusions

Among middle-aged and older people in Europe, there was a significant negative correlation between HGS and the risk of new-onset diabetes. In addition, the results suggest that relative HGS exhibited a slightly higher predictive ability on future diabetes than dominant HGS, and further researches are needed to explore the clinical significance of this modest difference. Our findings highlight that screening lower HGS in people aged 50 years or older may be worthwhile for diabetes prevention.

## Data Availability

The datasets generated and/or analysed during the current study are available in the Harmonized SHARE dataset and Codebook, Version E as of October 2019 developed by the Gateway to Global Aging Data, http://www.share-project.org/home0.html.

## Abbreviations

HGS: Handgrip strength
BMI: Body mass index
HR: Hazard ratios
95% CI: 95% confidence interval
SHARE: The Survey of Health, Ageing and Retirement in Europe International Diabetes Federation (IDF)
NRI: Net reclassification index
IDI: Integrated discrimination improvement
PURE: The Prospective Urban-Rural Epidemiology
CoLaus: Cohorte Lausannoise
KNHANES: Korea National Health and Nutrition Examination Survey
